# microRNAs in the Antitumor Immune Response and in Bone Metastasis of Breast Cancer: From Biological Mechanisms to Therapeutics

**DOI:** 10.3390/ijms21082805

**Published:** 2020-04-17

**Authors:** Marta Gomarasca, Paola Maroni, Giuseppe Banfi, Giovanni Lombardi

**Affiliations:** 1 Laboratory of Experimental Biochemistry & Molecular Biology, IRCCS Istituto Ortopedico Galeazzi, 20161 Milano, Italy; marta.gomarasca@grupposandonato.it (M.G.); banfi.giuseppe@fondazionesanraffaele.it (G.B.); giovanni.lombardi@grupposandonato.it (G.L.); 2 Vita-Salute San Raffaele University, 20132 Milano, Italy; 3 Department of Athletics, Strength and Conditioning, Poznań University of Physical Education, 61-871 Poznań, Poland

**Keywords:** breast cancer, bone metastases, miRNAs, NK cells, immune evasion, miRNA-based therapeutics

## Abstract

Breast cancer is the most common type of cancer in women, and the occurrence of metastasis drastically worsens the prognosis and reduces overall survival. Understanding the biological mechanisms that regulate the transformation of malignant cells, the consequent metastatic transformation, and the immune surveillance in the tumor progression would contribute to the development of more effective and targeted treatments. In this context, microRNAs (miRNAs) have proven to be key regulators of the tumor-immune cells crosstalk for the hijack of the immunosurveillance to promote tumor cells immune escape and cancer progression, as well as modulators of the metastasis formation process, ranging from the preparation of the metastatic site to the transformation into the migrating phenotype of tumor cells. In particular, their deregulated expression has been linked to the aberrant expression of oncogenes and tumor suppressor genes to promote tumorigenesis. This review aims at summarizing the role and functions of miRNAs involved in antitumor immune response and in the metastasis formation process in breast cancer. Additionally, miRNAs are promising targets for gene therapy as their modulation has the potential to support or inhibit specific mechanisms to negatively affect tumorigenesis. With this perspective, the most recent strategies developed for miRNA-based therapeutics are illustrated.

## 1. Introduction

Metastatic cancer is a main cause of death, and early diagnosis of the primary tumor, as well as the constant monitoring of disease progression and the patient’s response to therapies and surgery, are essential to efficiently manage subjects with cancer. The increasing knowledge of the biological mechanisms underlying the transformation and consequent metastatic transition has brought the discovery of new therapeutic targets and the development of more and more effective treatments that act on metastasis and/or on the primary tumor in order to limit the risk of metastasis.

Several circulating molecules, produced by the primary tumor, are involved in priming the receiving tissue that will potentially host the metastasis. This is a particularly critical phase because it allows the constitution of a favorable microenvironment in which cells express molecules able to recall the primary tumor cells and further stimulate them to express a migration favoring phenotype. Similarly, the phenotypic changes associated with the shift into a migrating phenotype, as well as the exposure to new environments (stroma, blood, metastasis-receiving tissue), expose this newly modified tumor cell to the checking of the immune cells. Although immunosurveillance is highly effective, primary and metastasizing tumor cells are able to develop strategies that allow the elusion of this control. Even in this case, several mediators are involved in the bidirectional crosstalk between tumor and immune cells [1,2]. A downstream effect induced by these signals is represented by the aberrant expression of microRNAs (miRNAs), which have been recognized as the main effectors in these processes. Furthermore, miRNA can be directly the effector of crosstalk, since they can be released by a cell in order to act in another site (the surrounding microenvironment, metastatic site, immune cells). In this way, the miRNA-mediated responses are involved in the overall process of transformation. Noteworthy, circulating miRNAs are promising markers for cancer diagnosis, prognosis, monitoring the treatment response, and as powerful tools for personalized approaches [3]. Since their biological relevance and the more and more deepened knowledge about the biological mechanisms associated with their expression in both physiological and pathological conditions, novel therapeutic approaches targeting or inducing miRNAs are, currently, under study.

The aim of this narrative review is to summarize the most recent findings relative to the miRNA-dependent regulation of immunosurveillance and immune-escaping of metastasizing breast cancer with a specific focus on bone metastasis.

### miRNA Biogenesis and Functions

miRNAs are a class of short single stranded (ss) non-coding RNAs with regulatory functions mainly related to the inhibition of gene expression. The hypothesis of the existence of regulatory RNA molecules [4] was confirmed after the discovery, in Caenorabditis elegans, of the short ssRNA lin-4, able to regulate the expression of the lin-14 gene throughout an antisense interaction [5]. Such short regulatory RNAs, named miRNAs, and the relative regulatory systems, have been discovered in all the living organisms, included viruses [6].

miRNAs are encoded from DNA sequences (i.e., miRNA genes) which are generally located within noncoding regions, introns and untranslated regions (UTRs) of protein-encoding sequences, and their expression is submitted to the same epigenetics regulation as for the protein-encoding genes, e.g., methylation, acetylation/deacetylation [7]. miRNAs are transcribed as a stem loop pri-miRNA, precursor by RNA polymerase II. This precursor undergoes different steps of maturation, first in the nucleus and then in the cytoplasm. The RNase activity of the nuclear enzyme RNase III Drosha DGRC8 cleaves the loop-flanking sequences, thus, releasing a pre-miRNA which is exported into the cytoplasm throughout Exportin 5. Here, another RNase, the RNase III Dicer, cleaves the loop region, leaving a double strand (ds) RNA that comprises a star strand, with one strand that will proceed further and will act as miRNA, and a passenger strand, that is degraded by the Dicer itself. In some cases, however, both strands are maintained and they both act as miRNAs; since these two miRNAs are generated by the same transcript, they are named in the same way with either a “3p” or “5p” suffix depending on the ending of origin. Finally, the mature miRNA is incorporated together with Argonaut (Ago)-2 protein within the RNA-induced silencing complex (RISC), which represents the effector of miRNA-based gene expression repression. A “seed region” of 6 nucleotides within the miRNA sequence (positions 2-to-5) recognizes in a more or less specific way the target mRNAs. The pairing between RNA molecules generate a dsRNA and the target mRNA is degraded within the RISC. The non-stringent specificity of the pairing and the short length of the seed region allow each miRNA to target even hundreds of different mRNAs [8]. Although a relatively recent discovery, the regulation of gene expression by miRNAs is fundamental for the correct functioning of the cell and its deregulation is associated with several diseases and can be also lethal, as demonstrated in Dicer-1 knock out mice, which is lethal early during the embryological development [9,10] in human embryos [11].

miRNAs can be expressed with either a cell type/tissue-specific expression or in a wider fashion and they can be released, either actively or passively, into the extracellular fluids, as free miRNAs or associated with proteins and lipoproteins or encapsulated into vesicles. Hence, miRNAs can be found in the blood, where they are relatively abundant (as well as virtually in all body fluids), and they can act on cellular targets in other districts. In such a condition, they can be used as markers to monitor a biological process and, since their expression is strikingly dependent upon multiple stimuli they can likely be used for personalized monitoring [12].

An abnormal miRNA expression is demonstrated in several disorders including tumors, and inflammatory and autoimmune diseases [3,12,13,14]. In general terms, miRNA expression is downregulated in tumors (since they are inhibitors of gene expression) compared to their non-tumor counterparts and a few miRNAs can mark a specific neoplasia [15]. Each step of the neoplastic transformation, for a given tumor, is associated with the deregulation of the expression of specific miRNAs as well as progression towards every different stage [16], included tumor growth, progression, metastasis, and development of drug resistance [17]. Moreover, a tumor can release miRNAs that can actively target other tissues to determine, for example, the appropriate microenvironment to host a metastasis [18].

Information about miRNAs can be found in databases such as miRBase (www.mirbase.org) [19] and MirGeneDB (http://mirgenedb.org) [20], which are continuously updated to an impressively growing number of discoveries around miRNAs.

## 2. Breast Cancer: Metastasis and Role of the Immune System

### 2.1. Breast Cancer

Breast carcinoma is the most common female cancer and, although important improvements in prognosis and in therapy have been achieved in recent decades, it remains one of the most frequent causes of death among women.

Based on hormonal status and on the expression of Ki67 (proliferation marker) breast cancer patients can be stratified at least in four distinct molecular subtypes. Luminal A (estrogen receptor, ER+ and/or progesterone receptor, PR+/− and Human Epidermal Growth Factor Receptor 2, HER2−; low Ki67 <14%), Luminal B (ER+ and/or PR+/−, HER2− with Ki67 ≥14% as well as ER+ and/or PR+/−, HER2+ with any Ki67), HER2 (ER−, PR−, and HER2+ with any Ki67) and Triple Negative Breast Cancer (TNBC, ER−, PR−, HER2− with any Ki67).

Among these subtypes, TNBC (further classified into six subclasses) represents the most heterogeneous subgroup; lacking the targets of specific hormonal therapies, most of the TNBC patients have a worse clinical outcome, a shorter relapses-free time and a strong possibility of developing bone, lung, brain and liver metastases. Therefore, TNBC is still one of the most fatal diseases for women, since it has poorer prognosis than other invasive breast cancers [21].

Breast cancer, as a heterogeneous disease, shows many different histological and molecular subtypes that differ in antigenicity, ability to escape immunotherapeutics and clinical treatments. Several studies have reported that each subtype also differs immunologically; for instance, a dense immune infiltration (T and B cells, macrophages and myeloid-derived suppressor cells, MDSC) has been mainly associated with high-grade histological subtypes, TNBC and HER2+ breast cancer, because it is less represented in the less aggressive Luminal A breast cancer [22,23]. Compared to ER− breast tumors, ER+ breast tumors are positively correlated to the presence of clusters of innate immune cells that favor a good prognosis: natural killer (NK) cells have antitumor activity in ER+ breast cancers, although their numbers decrease in advanced tumor stages [24,25,26].

### 2.2. Bone Metastasis

The transfer of neoplastic cells to distant sites is the most common cause of cancer-associated mortality; in particular, bone represents a main metastatic and recurrent site for breast and prostate cancer cells (~60%/70% of metastatic breast and prostate cancer patients metastasized to bone).

Bone has peculiar features that facilitate the engraftment and growth of neoplastic cells, acting as a fertile soil: mineral content, matrix composition, extreme rigidity, highly hypoxic environment, acidic pH, and high concentration of extracellular calcium. Bone metastasis represents also the major cause for morbidity due to skeletal-related events (SREs): pathologic fracture, severe pain, spinal cord compression, hypercalcemia, bone marrow aplasia, that impair quality of life and reduce survival of the patients.

Bone remodeling is carried out throughout the highly coordinated action of bone-forming osteoblasts and bone-resorbing osteoclasts; the presence of metastatic lesions in bone disrupts this fine balance and, consequently, disrupts the local microenvironment. In particular, breast cancer cells reaching the bone marrow activate a remarkable pro-resorptive response (osteolytic metastasis). In addition to directly stimulating osteoclasts, cancer cells indirectly induce osteoclastogenesis and bone destruction by altering the expression of many growth factors, cytokines, chemokines, and signaling pathways by osteoblasts and osteocytes. Parathyroid hormone-related protein (PTHrP), for example, supports osteoclasts formation by increasing RANKL expression in osteoblasts. In turn, the bone releases factors during excessive bone resorption (mediated by osteoclasts) such as Ca2+ and TGF-β, which are responsible for tumor growth. This is the “vicious cycle” that feeds itself and promotes metastatic growth [27].

In breast cancer, the period between the detection of the primary tumor and the recurrence of metastasis (metastatic latency) is variously long: in the ER− tumors, this latency takes about 5 years after diagnosis (short/medium latency), while in ER+ tumors the recurrence develops later within 8 and 10/15 years after diagnosis (long latency).

This dormancy reflects the capability of disseminated tumor cells (DTCs), or micrometastases, to evade treatments and to remain in a temporary growth arrest state after primary tumor resection [28], but the cells retain metastasis-initiating power that induces cell-escape from dormancy and macrometastasis development. In the dormant state, the cells are more resistant to almost all currently available therapies, as cytotoxic therapies preferentially target proliferating cells instead of cells that have left the active cell cycle [29,30].

Malladi et al. demonstrated that breast and lung carcinoma cells seeding in distant organs persist in a dormant state because of the capacity to escape from NK cells through the inhibition of various NK cell-activating ligands, and this overlaps with the entrance into a quiescent state. These dormant cells injected into immunocompromised mice (defective for both adaptive and innate immune responses) restart to grow out, indicating the importance of the innate immune system in controlling latency and, consequently, in the development of bone metastasis [31].

### 2.3. Breast Cancer and Immunity

Immune cells constitute an important component of the tumor microenvironment and are found in two compartments: in the stromal tissue, surrounding the tumor, and in the bulk of the tumor.

The identification and destruction of nascent tumor cells is a process termed cancer immunosurveillance and functions as an important defense against cancer. In overall immune response, innate immunity (first and non-specific step) participates actively, with a plethora of cells of myeloid lineage (dendritic cells, DCs, monocytes, macrophages, polymorphonuclear cells, mast cells), and innate lymphoid cells (NK), while adaptive immunity (second and specific step) depends on precise recognition of the antigen via B cell or T cell receptors.

In particular, the antigen presenting cells (APCs), macrophages, and DCs link the innate and the adaptive immunity, secrete cytokines that stimulate innate immune cells and present antigens to the T cells in the context of major histocompatibility complex (MHC) class II molecules.

The interplay between cancer and the immune system was summarized by Dunn and colleagues [32] under the name of immunoediting. The immunoediting process consists of three phases: elimination, equilibrium and escape. Elimination and equilibrium are the phases in which the immune system has the capacity to eliminate or has the ability to control tumor growth. The escape phase, on the other hand, in which tumor cells evade immune destruction, has been recognized as a hallmark of cancer due to its role in tumor progression and metastasis. In this phase, neoplastic cells, due to autonomous modifications, evade immune detection and destruction as well as induce an immunosuppressive microenvironment that alters the function of effector cells [26]. This process clearly describes the effects on cancer progression of tumor-associated immune cells that can be both positive and negative [33,34,35].

More recently, the attention was turned to the role of tumor-infiltrating immune cells (TIICs) in breast cancer. Tumor infiltrating lymphocytes (TILs), the most widely studied populations of TIICs, are reported as important prognostic factor in HER2+ breast cancer and in TNBC, as their presence is associated with enhanced overall survival (OS) and better response to treatments [36].

CD8+ cytotoxic T cells, among TILs, represent the class of lymphocytes that correlate with positive clinical outcomes and prognosis and, usually, infiltrate a large amount breast lesions [37]. This association with prognosis, however, was detected in ER− and in HER2+ breast tumors while not observed in ER+ breast cancers [38]. CD8+ T lymphocytes, together with NK cells, serve as the first-line of immune-defense.

Among T cells, the CD4+ regulatory T cells (Treg, CD4+, CD25+, FOXP3+) are potent immune suppressors that play important roles in maintaining homeostasis in the immune system. Treg cells are present as infiltrates in various cancers: pancreatic, melanoma, and breast cancers; in particular, in breast and pancreatic cancers, Treg cells are increased, in the tumor microenvironment, and also in peripheral blood [39]. The enhanced presence of Treg cells (FOXP3+) in breast tumor biopsies is linked to progressive disease and reduced relapse-free and OS, and may indicate that the presence of Treg cells promotes tumor progression by inhibiting immunosuppression [40].

Several studies suggest that TILs might also serve as a predictive biomarker of beneficial cytotoxic therapy and biomarkers of residual disease after neoadjuvant chemotherapy [41].

Another immune population that can regulate the interaction between cancer cells and immune system are the tumor-associated macrophages (TAM). Macrophages are very peculiar cells that, due to their strong plasticity, participate in both innate and adaptive immunity when stimulated by external stimuli. Microenvironmental signals induce the polarization of macrophages into distinct phenotypes M1 (classically activated) and M2 (alternatively activated), based on the main biological processes in which they participate. M2 macrophages, which account for most of the TAMs, play a role in establishing an immune environment that is permissive for tumor growth and spreading. TAMs exert immunosuppressive activity through the expression of an immune checkpoint receptor, called programmed cell death 1 (PD-1), which leads to the inhibition of cytotoxic TILs after binding with its ligand, the programmed cell death ligand 1 (PD-L1) [42].

## 3. microRNAs in Immune Escaping and Metastasis

Recently, miRNAs have emerged as master regulators of the molecular pathways involved in the sequential steps from tumorigenesis to progression and metastasis. More and more studies are, indeed, associating the deregulated expression of single miRNAs or patterns of miRNAs to the aberrant expression of oncogene and tumor-suppressor genes. Although the specificity of the binding miRNA-target mRNA, each miRNA is able to target several mRNAs and, consequently, the effect of each miRNA is comprehensively wide [3].

The immune system functions and the metastasizing process are themselves under the control of multiple miRNAs, whose knowledge is still far from complete. However, some mechanisms are currently understood, putting the basis for the development of new therapeutic strategies. Targeting the expression of immune function-regulating miRNAs, in immune cells, tumor cells can enhance their survival probability. As a response, deregulated tumor immunity can result in the release, as is for cytokines, of miRNAs from immune cells towards the target tumor cells, where they explicate their inhibitory action [43]. At the same time, miRNAs regulating different stages of the metastasis formation can favor this process, especially through the downregulation of tumor suppressor genes and the concomitant upregulation of oncogenes, for example preparing tumor cells to the migrating phenotype or even preparing the metastatic site to receive circulating tumor cells to initiate and establish metastasis.

### 3.1. miRNAs and Cancer-Related Immunity

Tumor microenvironment is associated with different types of immune cells, both from the adaptive and the innate immune system. The fine-tuned equilibrium between the two branches determines whether the immunity act as pro- or antitumorigenic factor, in a process called cancer immunoediting [44,45]. While, on one side, innate and adaptive branches of the immune system are able to eliminate neoplastic cells by recognizing tumor neo-antigens as non-self and grant effective immunosurveillance, on the other side, changes in the malignant cells, or either in the host immune cells, prevent the recognition of tumor cells by the immune system, failing in the elimination of tumor cells and, thus, allowing the tumor to escape the immune attack [32,46]. In this context, the crosstalk between tumor and immune cells is pivotal. miRNAs are proven key regulators of the miRNAs-immunity-cancer connection, as they are known to regulate both physiological and pathological processes, including cancer and inflammation [47]. Deregulation of miRNAs biogenesis or expression has been associated with tumor onset and progression, metastasis formation, and resistance to therapy in various solid tumors [44]. Tumor-associated miRNAs can either exert an oncogenic function, such as miR-155, miR-17-92 cluster, and miR-21, or have a tumor-suppressive role, i.e., miR15/16, let-7 family, and miR-34 family [48]. In functioning as a bridge between immune response and cancer, miRNAs regulate the recruitment and activation of specific immune cells within the tumor microenvironment, and target the specific cancer-related pathways in the immune cells that can lead to the secretion of immunosuppressive or immunostimulating factors by either cancer cells or immune cells, thus contributing to cancer development [44].

Tumor-associated miRNAs can either have a role in controlling the development and function of immune cells population within the tumor microenvironment or alter the expression patterns of the tumor cells resulting in a differential modulation of immune cell recruitment or immune cells recognition of the transformed cells, thus limiting the antitumor response. miRNAs involved in the regulation of tumor immunogenicity or in the antitumor immune response are identified as immune-modulatory miRNAs (im-miRNAs) and can be further classified as tumor suppressive im-miRNAs when they help in promoting the immunogenicity and the immune response and improve immunosurveillance, or oncogenic im-miRNAs, when they suppress the immune response and favor tumor immune escape [49].

**Figure 1:**
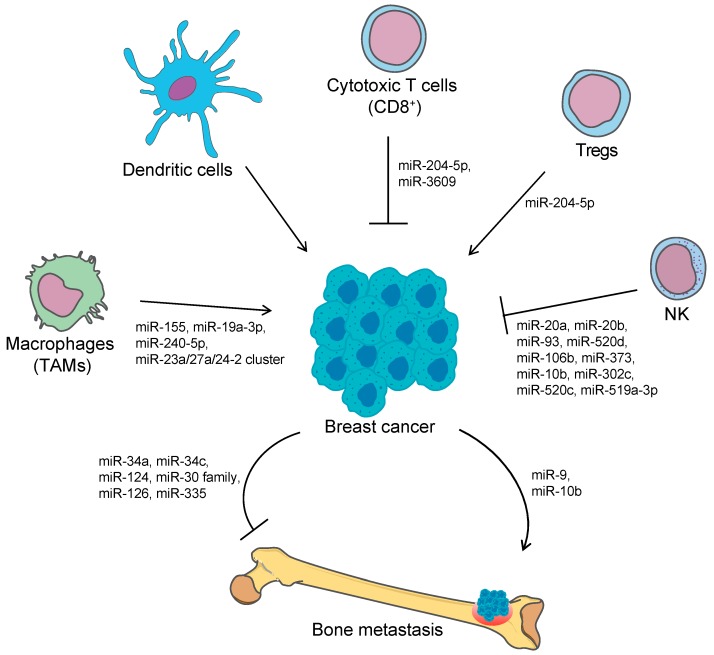
miRNAs involved in cancer-related immunity and bone metastasis in breast cancer. Interplay between immune cells that promote breast cancer growth and favor tumor microenvironment (arrows) and immune cells that inhibit breast cancer progression (dashed lines), with relevant miRNAs involved in the process. Other miRNAs are involved in the promotion or inhibition of bone metastasis of breast cancer. This figure was produced using Servier Medical Art available at https://smart.servier.com/. TAMs: tumor associated macrophages; Tregs: regulatory T cells; NK: natural killer cells.

miR-155 is a well-known oncogenic miRNA commonly overexpressed in many types of tumor, including breast cancer [50]. It has been reported that miR-155 regulates the antitumoral response in different subsets of immune populations in breast cancer. In a spontaneous breast cancer mice model, knock down of miR-155 in myeloid cells significantly accelerated tumor growth by impairing the activation of the TAM, increasing the production of pro-tumor cytokines and reducing the expression of activation markers, thus concurring in shifting TAM from a M1-phenotype to a M2-phenotype and creating an immunosuppressive microenvironment [51]. Similarly, the miR-23a/27a/24-2 cluster contributes to breast cancer progression modulating macrophage polarization [52]. Recently, miR-195 and miR-497 were found to downregulate the cluster of differentiation *CD274* (also known as PD-L1) in triple negative breast cancer cells, suggesting that miR-195/miR-497 influence tumor progression, inhibit the immune response and promote tumor immune escape [53] (Figure 1).

On the contrary, miR-19a-3p and miR-240-5p act as tumor-suppressive miRNAs in breast cancer. miR-19a-3p decreases the M2-like TAM population as it regulates the shift from the M2- to M1-phenotype of TAMs, by targeting the proto-oncogene *Fra-1* and its downstream signaling pathways, both in vitro and in vivo, and contributes to the inhibition of metastasis formation [54]. miR-240-5p regulates the expression of key genes involved in the immune pathways, including the expression of cytokines such as tumor necrosis factor (TNF), contributing in the remodeling and reprogramming of the tumor microenvironment. The upregulation of miR-240-5p correlated with a significant reduction of MDSCs, macrophages, and NK cells, as well as an increased number of CD4+ T cells, CD8+ T cells, and regulatory T cells in the tumor microenvironment. Additionally, the overexpression of miR-240-5p resulted in a significant alteration in the metabolic properties of cancer cells and suppression of tumor growth and metastasis in vivo [55].

Altogether, these studies demonstrated that the modulation of the expression of single or multiple miRNAs in either tumor cells or immune cells could lead to the activation of specific signaling pathways or different immune cells types in the tumor microenvironment, eventually altering the immune cell functions.

Table 1 summarizes the oncogenic and tumor-suppressive miRNAs involved in breast cancer progression.

#### 3.1.1. miRNA and Immune Checkpoint Regulation

Among the number of mechanisms adopted by cancer cells to evade antitumor immunity, there is the manipulation of the immune checkpoint.

Immune checkpoint proteins (ICPs) are pivotal factors in the regulation of immune responses and there are some immune checkpoint inhibitors, e.g., against PD-1/PD-L1 and cytotoxic T lymphocyte antigen 4 (CTLA-4), which showed promising therapeutic results, and their use was approved, for some cancer treatments, by the United States’ Food and Drug Administration (FDA) [85].

PD-1 receptor is found on the surface of many immune cells, while PD-L1, its major ligand, is expressed by several cell types, including cancer cells. PD-1/PD-L1 association inactivates T cells and blocks the immune system, therefore interfering with the PD-1/PD-L1 binding could be a promising strategy for clinical applications. The PD-1/PD-L1 blockade received approval from the FDA as a standard cancer therapy for solid tumors such as breast cancer (TNBC). PD-L1 is expressed in a quarter of all breast cancers and it was reported that high expression levels are associated with poor OS [86].

The regulation of PD-1/PD-L1 signaling occurs at different levels either through the crosstalk with other immune targets or with other signaling partners involved in tumor progression.

Various reports showed that numerous miRNAs could have modulatory roles in ICPs’ expression in different types of cancer: miR-15a/miR-15b [87], miR-34a [88], miR-200 [89], miR-424 [90], miR-513 [91], miR-138-5p [92], and miR-142-5p [93] regulate the expression of PD-L1. It has been reported that miR-28, miR-138, miR-4717 and miR-374b could regulate PD-1 expression and affect the immune status of T cells in some cancers, highlighting the important role of miRNA in reducing or enhancing T cell function [94].

Some miRNAs have been found to directly target the 3’-UTR of PD-1 or PD-L1 mRNA, while others miRNAs may regulate PD-1/PD-L1 indirectly via signaling molecules (such as IFN-γ, PTEN, mTOR, STAT and others) [95]. In TNBC cells, miR-195 and miR-497 regulate CD274 (PD-L1) expression by direct targeting [53].

Li et al. showed that miR-3609 expression was lower in resistant cells (MDA-MB231 and MDA-MB468) than in more sensitive breast cancer cells (MCF-7), the exact opposite for PD-L1 expression. Moreover, the authors correlated the low expression of miR-3609 and the high expression of PD-L1 with poor prognosis in breast cancer patients. These authors suggested that the restoration of miR-3609 may sensitize breast cancer cells to adriamycin through the blocking of PD-L1 expression and, therefore, miR-3609 could be used as a chemotherapeutic target for breast cancer [74].

CTLA-4, member of the CD28 family, is another ICP [96] and, with its receptors CD80 and CD86, is involved, on the one hand, in the suppression of the effector T cells activity by the antigen-presenting cells, such as dendritic cells and macrophages, and on the other hand in the activation of Treg cells [97]. Therefore, the blockade of the inhibitory effects of CTLA-4 on T cells-mediated immune response is used to abolish immunological tolerance in the treatment of some types of cancer [98,99].

CTLA-4, expressed and functional on human breast cancer cells, influences the maturation and function of DCs in vitro; Chen et al. reported that the blockage of CTLA-4 restore DCs function as antigen-presenting cells and T cells activation, but also inhibited the biological activity of breast cancer cells. This study supported the clinical application of CTLA-4 blockade in breast cancer therapy [100].

In ovarian cancer cell lines and in ovarian cancer tissues, it was reported that the expression of miR-424 negatively correlated to the expression of CTLA-4/CD80 and PD-L1, and that the restoration of miR-424 re-established T cells activity, reverted chemoresistance and increased free survival time [90]. Low expression of the miR-424-3p was a significant predictor for prostate cancer aggressiveness and outcome, and this was in close correlation with CTLA-4 expression [101].

High expression levels of miR-195 and miR-16 were inversely correlated with PD-L1/PD-1 and CD80/CTLA-4 expression, and the blocking of the PD-L1 immune checkpoint could enhance radiotherapy via activation of the T cell response at the tumor microenvironment in prostate cancer, revealing biological and functional interactions between immunotherapy and radiotherapy through the miR-195/-16 family regulatory cascade [102].

miR-155 has been found to directly target the 3′-UTR of CTLA-4. In the miR-155 conditional knockout mice model, it was reported that TAM activation was reduced and the proliferation of injected melanoma cells increased. These results suggest that the increase of miR-155 expression could be useful to improve anticancer immunotherapies [103].

To date, the regulation of CTLA-4 by miRNAs is still poorly understood in breast cancer.

Manipulation of immune checkpoint protein expression by miRNA-based therapeutics combined with anti-immune checkpoint drugs may represent an improvement of cancer treatments.

#### 3.1.2. NK-Mediated Immune Surveillance and miRNAs Involved in Immune Escaping

The complexity of tumor microenvironment is infiltrated with innate immune components aimed at eradicating the transformed cells. As initially most of the attention was drawn on the role of T cells in recognition of cancer cells, other immune cells, such as the NK, are able to detect and eliminate transformed cells [104].

In this context, NK cells detect malignant or damaged cells through the recognition of molecules that are upregulated under cellular stress response [104]. These molecules are referred to as stress-induced ligands, and in the human genome, they exist in eight different forms: the major histocompatibility complex class I–related molecules A and B (MICA and MICB), and the unique long 16 binding proteins 1-6 (ULBP1-6) [105]. All these ligands are recognized by a unique receptor, the NKG2D receptor, which is expressed on several immune cells, such as NK, cytotoxic T cells and other T cell subsets, from both the innate and the adaptive immune system [106]. The NKG2D receptor is considered the genuine activating receptor on NK cells, able to activate NK cell cytotoxic activity and production of cytokines, as well as a co-stimulatory receptor on T cells by inducing their differentiation and expansion [105]. Thus, NKG2D alone, through the recognition of stress-induced ligands or NKG2D-ligands (NKG2DL) differentially expressed under different cellular stress conditions, can recognize cells suffering from DNA damage, infection, oxidative stress, excessive proliferation or oncogene activation, all markers of cellular stress or malignant transformation, facilitating the immune surveillance [105]. In this way, NK cells exert a strong antitumor activity via the NKG2D/NKG2DL axis.

Nevertheless, transforming cells have evolved a plethora of strategies to downregulate the expression of the NKG2DL, both at post-transcriptional and post-translational levels, in order to evade from the NKG2D-mediated surveillance, which is a prerequisite for immune escape. Indeed, in order to efficiently eliminate the transforming cells, the NKG2D on the immune cells have to recognize the ligands before immune-editing takes place, thus preventing the escape from immune recognition [105]. The ability of tumor cells to evade immune surveillance has been associated with a poor prognosis and a reduced outcome of immunotherapies in several types of cancers [104]. Thus, targeting the NKG2DL/NKG2D axis can be considered a powerful strategy for cancer therapy.

The evasive strategies developed by tumor cells to reduce the expression of the NKG2DL and, thus, to evade NK-mediated recognition, comprise epigenetic modifications including DNA methylation and histone deacetylation, promotion of alternative splicing or alternative adenylation of mRNA resulting in the expression of different isoforms of the ligands, or downregulation of the transcription of specific mRNA by the action of miRNAs. Other mechanisms prevent the ligands from being exposed on the cell surface, such as intracellular retention, cell internalization and degradation by the proteasomal pathway, or release from the cell membrane in a process referred as shedding, by both proteolytic cleavage of the extracellular domain or by incorporation into exosomes [104,105].

The expression of the NKG2DL on tumor cells is often associated with a better prognosis. In colorectal cancer, high expression levels of MICA significantly correlated with a good prognosis [107]. Similarly, it has been recently found that increased expression of MICB could be considered a good prognostic factor for OS for patients with breast cancer [64]. Interestingly, only the membrane-bound form of the ligands has been associated with a positive survival prognosis for patients [108].

Several miRNAs have been reported to regulate the expression of the NKG2DL (Figure 1). MICA and MICB expression are regulated by different miRNAs, namely miR-20a, miR-93, miR-520d, miR-106b, and miR-373, with oncogenic functions in several human cancer cells, including prostate, kidney and breast cancer cell lines. Furthermore, treatment with the specific miRNA antagonists resulted in an increased expression of MICA/B followed by the enhancement of NK-mediated killing [56]. In hepatocellular carcinoma, the miR-25-93-106b cluster, paralogue of the miR-17-92 cluster [109], was found to suppress MICA expression, leading to evasion of the NKG2D-medated response of NK both in vitro and in an in vivo cell-killing model [110]. Similarly, inhibition of miR-20a, miR-93 and miR-106b in glioblastoma cell lines has led to the upregulation of NKG2DL expression and, consequently, to the increased susceptibility to NK-mediated cytotoxicity [57]. The metastasis-associated miR-10b directly targets the 3′-UTR of MICB, but not MICA, and downregulates its expression in several cancer cell lines, impairing the ability of NK cells to recognize and eliminate tumor cells. In contrast, antagonizing miR-10b function restored the NKG2D-mediated cytotoxicity of cancer cells in vitro as well as increased tumor clearance in vivo, directly linking metastasis formation and immune evasion [58].

The ULBPs are also regulated by the action of miRNAs. miR-34a and miR-34c act as tumor suppressive miRNAs controlling the expression of ULBP2, by directly targeting the ULBP2 3′-UTR, in human malignant melanoma. The overexpression of the two miRNAs leads to upregulation of ULBP2 expression while their miRNA mimics inhibit the receptor expression, reducing the recognition of tumor cells by NK. Interestingly, the miR-34 expression levels are upregulated by the tumor suppressive protein p53, further proving the miRNA-immunity-cancer connection [111]. miR-302c and miR-520c negatively regulated the expression of MICA/B and ULBP2 upon 1-α,25-dihydroxyvitamin D3 (1,25(OH)2D3) treatment in leukemia (Kasumi-1) and breast cancer (MDA-MB-231) cell lines, by directly targeting the 3′-UTR, and their inhibition resulted in an increased resistance to NK cell killing activity [63]. Additionally, it has been proposed that tumor-suppressive miRNAs miR-140-5p, miR-409-3p, miR-433-3p and miR-650 regulate ULBP1 expression, thus modulating the NK cell response in different cancer cell lines [112].

Several studies reported the involvement of miRNAs in the regulation of the NKG2DL expression in breast cancer, contributing to the tumor immune escape. Members of the miR-17-92 cluster, miR-20a, miR-20b, miR-93, and miR-106b, downregulated the expression of MICA/B and ULBP2 in breast cancer cell lines, affecting the capacity of NK cells to recognize and eliminate the tumor cells. In particular, the miRNAs directly targeted the MICA/B 3′-UTR, while repressing ULBP2 expression by inhibiting the MAPK/ERK signaling pathway [64]. miR-20a was also found to modulate the expression of MICA/B in breast cancer stem cells: overexpression of miR-20a resulted in the downregulation of MICA/B which consequently reduced the susceptibility to NK-mediated cell lysis and enhanced the metastatic potential, thus favoring tumor progression and metastasis formation [65]. In addition, miR-519a-3p impaired NK-mediated breast cancer cell cytotoxicity by downregulating the expression of MICA and ULBP2. Interestingly, miR-519a-3p additionally targets TRAIL-R2, caspase-7 and caspase-8, conferring resistance to apoptosis mediated by TNF-related apoptosis-inducing ligand (TRAIL) and Fas ligand as well as granzyme B/perforin. These data strongly suggest that miRNAs regulate tumor progression and evasion from immunosurveillance by acting simultaneously at multiple levels [66].

Importantly, the NKG2DL-targeting miRNAs are epigenetically regulated: their expression can be inhibited by treatment with histone deacetylase inhibitors (HDACi). In particular, miRNAs belonging to the miR-17-92 cluster were downregulated by HDACi leading to the upregulation of MICA/B and ULBP2 and enhancing tumor cell lysis by NK in different hepatocarcinoma and breast cancer cell lines [64,113]. These studies suggest that treatment of patients with HDACi might lead to a combinatorial effect of tumor inhibition and increased immune activation.

### 3.2. miRNA in Metastasis

Besides acting as tumor suppressors or oncogenes, miRNAs also regulate metastasis formation and progression. These miRNAs are termed metastamiRNAs and act through a different mechanism to promote or inhibit metastasis: they interfere with migration and invasion of tumor cells, regulate epithelial-mesenchymal transition (EMT), alter functions and properties of cancer stem cells and modulate the tumor microenvironment [59] (Table 1).

One of the best known pro-metastatic miRNAs is miR-10b. This miRNA is overexpressed in several highly metastatic and invasive human cancer types, including breast cancer, pancreatic cancer and glioblastoma. Mechanistically, it has been shown to downregulate several tumor suppressor and metastasis suppressor genes. In particular, miR-10b is known to target directly the transcription factor TBX5, that in turn leads to the repression of the tumor suppressor genes *PTEN* and *DYRK1A*, the gene encoding for neurofibromin, which negatively regulates Ras and thus promotes proliferation, and *KLF4*, which promotes migration and invasion, altogether contributing eventually to the progression of tumorigenesis and metastasis [59,60]. In breast cancer, overexpression of miR-10b promotes tumor cells invasion and metastasis, both in vitro and in vivo, through the inhibition of the transcription factor homeobox D10 (HOXD10) and the consequent upregulation of the pro-metastatic gene RhoC. Conversely, inhibition of miR-10b with an antagomiR inhibits formation of lung metastasis in a mouse mammary tumor model [61,62].

Similarly, the metastasis-promoting miRNA miR-9 was found to promote metastatic ability in breast cancer by targeting multiple metastasis suppressors including E-cadherin, involved in EMT, and leukemia inhibitory factor receptor (LIFR), that suppress metastasis formation by inactivating the Hippo signaling pathway and was recently reported to be a breast cancer suppressor of bone metastasis [67,68,69].

Tavazoie and coworkers identify miR-126 and mir-335 as metastasis suppressor miRNAs. These miRNAs are downregulated in breast cancer and the restoration of their expression in highly metastatic breast cancer cell line MDA-MB-231 inhibits the formation of metastasis to the lung and bone in vivo. Induction of miR-126 reduces the overall growth and proliferation of the tumor, while restoration of miR-335 inhibits cell invasion, migration and metastasis by targeting the transcription factor SOX4 and the extracellular matrix protein tenascin C [75]. Additionally, miR-126, in pair with miR-126*, exert an antitumor role also through the downregulation of chemokines involved in the recruitment of mesenchymal stem cells and monocytes at the tumor microenvironment, and thus contrasting breast cancer migration, invasion and metastasis [76].

Another mechanism by which miRNAs regulates metastasis formation is by modulating the EMT. miR-200 family members, known to be associated with tumor metastasis and poor patient prognosis, together with miR-205, were found to inhibit EMT by targeting the transcription factors ZEB1 and ZEB2 involved in the induction of EMT [77]. In addition, overexpression of miR-200 in intravenously injected mouse mammary tumor cells resulted in the formation of macroscopic metastasis in lung and liver in mice, indicating that miR-200 favor colonization of tumor cells at distant organs [78]. The miR-200 family not only inhibits EMT but also promotes mesenchymal-epithelial transition (MET), the reverse process of EMT that contributes to metastatic colonization in some types of cancer [114]. The tumor suppressor miR-190 was found to suppress metastasis formation in breast cancer, by directly targeting SMAD2 and antagonizing TGFβ-induced EMT, creating a feedback-loop with TFGβ/SMAD2 signaling in controlling breast cancer EMT and metastasis [79].

Overexpression of the miR-200c/141 cluster, also a member of the miR-200 family, was found to upregulate SerpinB2, also known as plasminogen activator inhibitor type 2 or PAI-2, in the MDA-MB-231 breast cancer cell line. SerpinB2 is overexpressed in different tumor tissues, correlates to poor prognosis in primary breast cancer and other solid tumors, and promotes tumorigenesis and metastasis. Indeed, overexpression of miR-200c/141 in a xenograft mouse model led to metastasis in the lung and lymph nodes, while siRNA-mediate silencing of SerpinB2 reverted the ability of miR-200c/141 to induce metastasis [70].

In addition, miRNAs involved in the regulation of the Wnt/β-catenin signaling pathway play a crucial role in modulating EMT and tumor metastasis [115]. miR-374a is highly expressed in metastatic breast cancer and it downregulates epithelial markers, such as E-cadherin, and upregulates mesenchymal markers, such as N-cadherin and vimentin, thus promoting EMT. Additionally, miR-374a has a role in activating the Wnt/β-catenin pathway by increasing the nuclear translocation of β-catenin and directly targeting negative regulators of the signaling pathway, such as PTEN or WNT5A [71]. Other tumor suppressor miRNAs that inhibit breast cancer migration, invasion and metastasis through the modulation of the Wnt/β-catenin signaling pathway are miR-148a and miR-340. miR-148a is downregulated in breast cancer cells and tissue and it is known to target WNT1, a ligand of the Wnt/β-catenin pathway [80]. miR-340 is remarkably downregulated in the highly metastatic breast cancer cell line MDA-MB-231 and potentially targets c-MYC and CTNNB1 (the gene encoding for β-catenin), and ROCK1, involved in the Wnt/β-catenin-dependent and -independent signaling pathways, respectively [81].

Interesting also, regulation of miRNA biogenesis modulates metastasis progression. For example, regulation of the expression of a key enzyme of miRNA biogenesis Dicer by miR-103/107 and miR-630 promotes metastasis, through the alteration in the expression of metastasis suppressor miRNAs, such as miR-200 family members [72,73]. Dicer can also be epigenetically downregulated by hypoxia, leading to the downregulation of miR-200 and consequent deregulation of ZEB1 [116].

In recent years, miRNAs are gaining increasing interest as potential biomarkers as they possess all the required characteristics of a biomarker, such as noninvasiveness, high sensitivity, specificity and predictivity, and translatability [117]. Some miRNAs have been detected in the circulation associated with metastatic breast cancer, suggesting their potential role as biomarker for discrimination between metastatic and non-metastatic breast cancers, as recently reviewed in [3]. For instance, elevated circulating levels of miR-10b, miR-155 and miR-34a were found in patients with metastatic breast cancer compared to patients with primary breast cancer without metastasis [118]. To this, it was subsequently found that circulating levels of miR-10b and miR-373 were elevated in patients with lymph node positive metastasis compared to patients with no metastasis or healthy control, increasing their potential as biomarkers [119]. However, circulating miR-10b has been found as a potential biomarker for different metastasis sites other than lymph nodes, such as brain and bone, thus indicating that it is not a reliable biomarker for site-specific metastasis [120].

In other studies, plasma levels of miR-21, miR-23b, miR-190, miR-200b, and miR-200c, all related to metastasis, EMT or tumor dormancy, were evaluated in early breast cancer patients demonstrating that they were predictive for disease recurrence and could be considered significant prognostic and diagnostic biomarkers in breast cancer [121,122].

Other metastamiRNAs involved in the regulation of metastasis progression found in tissue have been also found elevated into circulation. Circulating levels of the miR-200 family members, together with miR-203, miR-210, miR-375 and miR-801 were found elevated in patients with increased circulating tumor cells [123]; increased blood and tissue levels of miR-105 levels were associated with metastatic progression of early-stage breast cancer compared to non-metastasizing breast cancer [124]. Similarly, differential expression levels of miR-17 and miR-155 were found between metastatic and non-metastatic breast cancer [125]. Many other miRNAs have been identified as potential biomarkers for both primary and metastatic breast cancer, which have been reviewed elsewhere [3,120]. Nevertheless, it is worthy to highlight the importance and the potential of circulating miRNAs as biomarkers for the discrimination between metastatic and non-metastatic breast cancer that could contribute to a more precise and personalized therapeutic approach for the patients.

#### miRNA in Bone Metastasis from Breast Cancer

It is known that the most common site for breast cancer metastasis is bone and metastasizing to this site is specifically regulated, among several factors, by miRNAs. Understanding how miRNAs regulate bone metastasis from breast cancer would improve the prognosis and OS of breast cancer patients. In recent years, the number of miRNAs inhibiting or promoting breast cancer bone metastasis has increased. Here we report some examples (Figure 1). miR-34a was identified as a suppressor of osteoclastogenesis, bone resorption and bone metastatic niche. It targets the pro-osteoclastogenic transforming growth factor-β-induced factor 2 (Tgif2), and it is downregulated during osteoclast differentiation. It was demonstrated that transgenic mice overexpressing miR-34a in pre-osteoclasts resulted in reduced ovariectomy-induced osteoporosis and reduced breast and skin cancer bone metastasis [82]. Similarly, the reduced expression of miR-124 in metastatic bone tissue from breast cancer is associated with aggressive clinical characteristics accompanied with shorter bone metastasis-free survival and OS. Restoration of miR-124 in breast cancer cells resulted in the suppression of bone metastasis in vivo through the targeting of intrleukin-11 (IL-11) and the consequent inhibition of osteoclast progenitor cells differentiation and survival [83].

Breast cancer bone metastasis suppressors are the miR-30 family members, miR-30a/e, whose low expression levels in primary breast cancer associated with poor relapse-free survival. miR-30 overexpression in ER−/PR− breast cancer cells resulted in the inhibition of tumor cells invasiveness and restoration of bone homeostasis in vitro, as well as reduction of bone metastasis in vivo [84]. Altogether, these studies demonstrated how miRNAs could be used for therapeutic purposes to attenuate bone metastasis from breast cancer.

## 4. miRNA-Based Therapeutics

**Figure 2:**
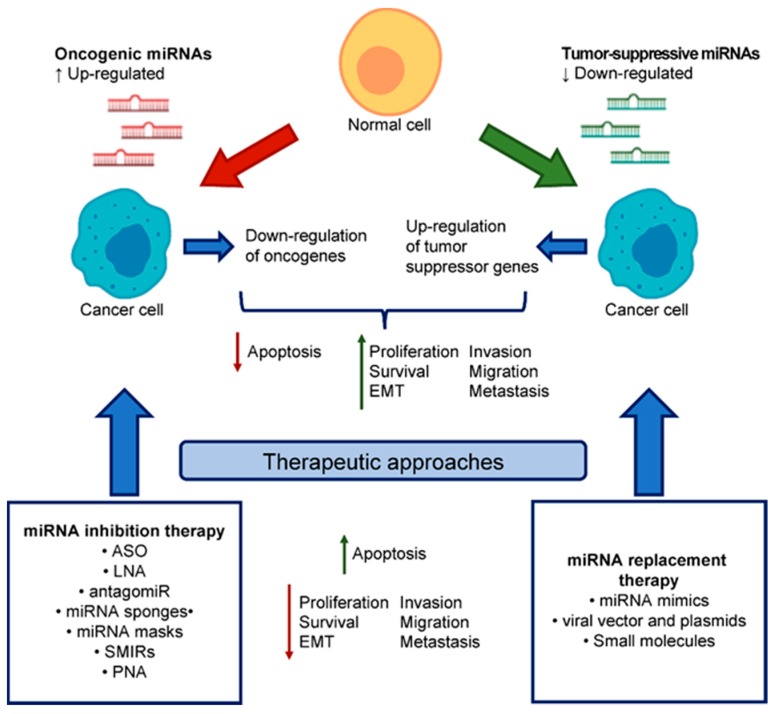
Role of miRNAs in cancer and approaches for miRNA-based therapies. Aberrant miRNAs expression is a common finding in cancer. Upregulation of oncogenic miRNAs determines a general downregulation of their tumor suppressor gene targets, while the downregulation of tumor-suppressive miRNAs induces the upregulation of the target oncogenes, altogether supporting the cancerous cell to block apoptosis and promote proliferation, EMT, invasion, migration and metastasis. Therapeutic approaches targeting miRNAs may consist of two strategies. The miRNA inhibition therapy is based on the use of different types of miRNA inhibitors to target upregulated oncogenic miRNAs, leading to the consequent upregulation of tumor suppressor genes. The miRNA replacement therapy, on the other hand, is based on the reintroduction of the downregulated tumor-suppressive miRNA function for the consequent downregulation of the target oncogenes. The effects of miRNA-based therapies contribute to promote an increase in cell death and counteraction of tumor development, eventually leading to the restoration of a more physiological phenotype of the malignant cell.

Considering the increasing number of miRNAs aberrantly expressed in cancer, and other diseases, miRNAs have emerged as appealing targets for therapeutics in the management of cancer. According to whether miRNAs are downregulated or upregulated in cancer, an effective therapy should be based on two different strategies: the reintroduction of miRNA function with the so-called miRNA replacement therapy or the inhibition of an overexpressed miRNA with the miRNA inhibition therapy, respectively (Figure 2). With these two strategies, it is possible to modulate the phenotype of malignant cells and contrast the progression, dissemination and metastasis formation of cancer. It is worth noting that the potential of miRNA-based therapeutics is based on the concept that a single miRNA can modulate multiple oncogenes and oncogenic pathways usually deregulated in cancer, thus the targeting of a single miRNA acts on multiple levels, amplifying the effect [126].

### 4.1. miRNA Replacement Therapy

Tumor-suppressive miRNAs are usually downregulated in cancer cells, consequently leading to the upregulation of the target oncogenes and the promotion of tumorigenesis. miRNA replacement therapy consists of the reintroduction of the tumor-suppressive miRNA to restore the lost function. In this aim, several agents can be used: e.g., small molecules, miRNA mimics, and DNA plasmids encoding a miRNA gene that alters epigenetically the endogenous expression of the miRNA [127].

Small molecules, used for the restoration of miRNA function, consists of hypomethylating agents or enoxacin that induce miRNA expression in a non-specific manner by enhancing miRNA synthesis and processing [126,128].

miRNA mimics, on the other hand, are far more used and studied. They are small synthetic RNA duplexes, usually chemically modified, bearing the same sequence of the tumor-suppressive miRNA, that are loaded into the RISC complex and processed to promote the downstream inhibition of the mRNA target [126]. Restoration of gene function is commonly used in gene therapy, where a protein-encoding gene needs to be reinserted in the target cells. For this purpose, usually DNA plasmids or viral vectors are used; however, this approach is limited by several drawbacks, such as vector size, the inefficient delivery to target cells or tissue, and the nuclear localization to exert their function. On the contrary, the great advantage of miRNA mimics is that they are substantially smaller, need to act in the cytoplasm of the cells to be biologically active, can be easily systemically delivered with delivery systems already developed for siRNAs, and nonspecific off-target effects are unlikely [129]. Additionally, it has been reported that mouse studies that involved the delivery of miRNA mimics did not show any adverse events associated with the compounds and the delivery to normal tissues was tolerated, suggesting that miRNA mimics do not exert any cytotoxic effects for normal tissue [129].

Even though the application of miRNA mimics for miRNA replacement therapies are very promising, one of the greatest challenges for their clinical application is represented by the development of the most suitable delivery system to afford an effective, efficient and specific delivery. The ideal delivery system should (i) prevent degradation of the miRNA cargo in the bloodstream, (ii) allow efficient targeting and distribution to the target tissue/cell, (iii) facilitate cellular uptake, (iv) avoid induction of the immune response, and (v) be biodegradable and biocompatible [130]. So far, the most commonly used delivery systems are divided in two categories: viral vectors, that are generally immunogenic, and non-viral vectors, that comprise polymeric vectors, lipid-based carriers, and carriers made of inorganic materials [131,132]. For instance, a miR-34a synthetic mimic was successfully incapsulated into lipid-based vehicles in a non-small-cell lung cancer mouse model, resulting in the accumulation of the miR-34a and the downregulation of the miRNA targets in the tumor cells, when administered both locally or systemically, without showing any immunogenicity [133]. Another group employed a chitosan nanoplex, a cationic polymer with high specificity for binding negatively charged nucleic acids, to deliver a miR-200c mimic in breast cancer cells, resulting in a decrease in angiogenesis, EMT, invasion and metastasis, and increased apoptosis. Even though the in vivo application of the chitosan/miR-200c nanoplex is missing, it is interesting how the effect of this nanoparticle is cell-type dependent, suggesting a possible differential effect according to the tumor type [134].

Thus, even though the proof of the effectiveness of miRNA mimics in restoring tumor-suppressive miRNAs functions is countless, it is of enormous importance to identify and develop an appropriate delivery system to upgrade their potential use in clinic.

### 4.2. miRNA Inhibition Therapy

Oncogenic miRNAs are overexpressed in cancer and their inhibition may contribute to the restoration of the normal expression and function of the target tumor suppressive genes. miRNA inhibitors are usually single-stranded oligonucleotides, complementary to the sequence of the oncogenic miRNA and chemically modified to enhance the affinity with the complementary miRNA. The anti-miRNA oligonucleotides are designed to bind to the guiding strand of the mature miRNA by Watson–Crick base pairing. The chemical modification of the anti-miRNA oligonucleotides, especially for the RNA-based ones, is necessary also to prevent further processing of the miRNA, as upon annealing they would recreate a dsRNA similar to the pre-miRNA [135]. Upon binding, they trap the endogenous miRNA in a configuration that cannot be recognized by the RISC complex, or lead to the degradation of the endogenous miRNA, resulting in the functional inhibition of the miRNA [126]. Among the miRNA inhibitors, there are antisense anti-miRNA oligonucleotides (AMOs), locked nucleic acid (LNA) anti-miRs, antagomiRs, miRNA sponges, miRNA masks, and small molecule inhibitors of miRNAs (SMIRs).

AMOs are single-stranded, chemically modified antisense oligonucleotides of 17–22 nucleotides in length, complementary to the targeted miRNA, that bind the mature miRNA and act as competitive inhibitors, thus impeding the interaction with the specific target mRNA [136].

AntagomiRs are single-stranded RNA molecules, 23-nt in length, complementary to the miRNA of interest, and both chemically modified and cholesterol-conjugated. These modifications increase stability and protect from degradation, while the conjugated cholesterol moiety contributes to produce positive physiological effects: a decrease in plasma cholesterol levels was detected after administration of antagomir-122 in mice [126,137]. Intravenous administration of antagomiRs against miR-16, miR-122, miR-192 and miR-194 reduced the endogenous expression levels of the corresponding miRNAs in several organs, including liver, lung, kidney, suggesting a poor tissue specificity. Importantly, however, antagomiRs are not immunogenic [137].

LNA, belonging to the family of AMOs, are oligonucleotides in which the ribose is modified with a methylene bridge connecting the 2′-O and the 4′-C atoms, locking the ribose ring in the 3′-endo conformation. This modification confers to the LNA high thermal stability and affinity for the complementary miRNA, higher solubility in aqueous moieties and, most importantly, increased metabolic stability for the delivery in vivo [126]. LNAs have been broadly used in in vitro studies for miRNA functional studies, revealing their potential as miRNA inhibitors. Pharmacokinetic and pharmacodynamic studies conducted in mice and monkeys on the LNA-anti-miR-221 revealed a short half-life, good tissue bioavailability, minimal excretion in the urine, and long-lasting detectability (up to 3 weeks) in mice vital organs and xenografted tumors, indicating the potential suitability of LNA anti-miRNAs for clinical applications [138].

miRNA sponges are vectors containing multiple, tandem binding sites to the miRNA targets that compete with the endogenous miRNAs for the mRNA binding [139], which were successfully used for the inhibition of miR-9 to suppress metastasis formation by breast cancer cells [67], while miRNA masks selectively inhibit the interaction between miRNA and its mRNA target, by masking the miRNA binding site on the mRNA target thus preventing its repression [140].

Finally, SMIRs are small molecules drugs that modulate miRNA activity by blocking miRNA biogenesis or by preventing miRNA–mRNA interaction [128]. Their use as anti-miRNA therapeutics is particular appealing as their development would require shorter times of production and approval, and reduced costs of overall production, compared to protein- or oligonucleotide-based drugs, thus shortening the effort for their translation from bench to bedside [141].

It is clear that the use of different types of miRNA inhibitors to suppress overexpressed oncogenic miRNAs is a promising and robust strategy for cancer treatment. Nevertheless, many challenges still need to be solved, including the optimization of the delivery systems, the improvement of their stability and the extensive understanding of their possible off-target effects, before their actual application in clinics.

### 4.3. PNA: Novel Class of MiRNA Inhibitors

Peptide nucleic acids (PNA) have recently emerged as a novel class of miRNA inhibitors. PNA have been described for the first time by Nielsen et al. and are DNA analogues in which the sugar phosphate backbone has been replaced by N-(2-aminoethyl)glycine units [142]. They are capable of efficiently hybridize with complementary DNA and RNA in a sequence-specific manner by forming Watson–Crick double helices [143]. PNAs have been initially proposed for antisense (by binding target mRNA) or antigene (by binding target gene) therapy. Following strand invasion, PNAs can form duplex or triplex with the double stranded DNA [144]. PNAs possess higher affinity for RNA rather than DNA, are more specific and, due to their unnatural backbone, are resistant to the action of DNases, RNases and proteases, all features that make PNAs optimal candidates as miRNA inhibitors [145]. In vitro studies revealed that anti-miRNA PNA are more effective inhibitors compared to other DNA-based antisense oligonucleotides, are not cytotoxic at high concentrations, show long lasting effect up to 9 days and are considerably stable at storage temperature [146].

As PNA are synthetic molecules, they can be easily modified to improve the affinity and the specificity for the target RNA or to achieve better cellular delivery and permeation. Indeed, one of the greatest challenges of PNA is their poor cell permeation in eukaryotic cells because of the neutral backbone [135]. Several delivery systems have been employed to improve cellular uptake, such as liposomes, microspheres, or wrapping the PNA in pseudovirions [147,148]. However, one of the most accredited strategies is the conjugation of the PNA to a cell-penetrating peptide (CPP), small peptides that possess self-penetration abilities and permit intracellular uptake of the conjugated molecules [149]. Several CPPs have been used to improve PNA permeability, including R6-penetratin, a peptide of four lysine residues, Tat, transportan, an eight-residues poly-arginine peptide (R8), or the nuclear localization sequence (NLS), which allows intracellular uptake and nuclear localization, particular useful for antigene PNAs [146,150,151,152].

Brognara and coworkers designed a polyarginine-conjugated PNA targeting miR-221 (R-pep-PNA-a221) that showed high affinity for the targeted miRNA and efficient cellular uptake, while the unmodified counterpart showed very poor cellular permeation. Importantly, R-pep-PNA-a221 treatment of breast cancer cell lines led to downregulation of miR-221 and the upregulation of the tumor suppressive target gene p27^kip1^ [151]. Another example of PNA anti-miRNA efficiently inhibiting miRNA function is the polyarginine-conjugated PNA anti-miR-210 that downregulated miR-210 expression in K562 cells and inhibited erythroid differentiation [153]. The same R-pep-PNA-a221 tested in breast cancer cells produced the same effect in glioma cells, leading to the downregulation of the miR-221 oncogene target p27^kip1^ [154], while co-administration of R-pep-PNA-a221 and a R8-conjugated PNA anti-miR-155 promoted apoptosis and reverted the drug-resistance phenotype of glioma cells [155].

Besides numerous studies revealing the usefulness of anti-miRNA PNA in vitro, several studies reported the potential application of anti-miRNA PNA also in vivo. PNA targeting miR-155 efficiently blocked miR-155 both in LPS-activated primary B cells and in mice, and led to upregulation of several miR-155 target mRNAs [150]. Gupta et al. developed a modified γPNA to ensure higher RNA binding affinity, solubility and enhanced biocompatibility: the γPNA targeting miR-210, a miRNA involved in the tumor cell adaptation to hypoxia, was encapsulated in poly(lactic-co-glycolic acid) (PLGA) nanoparticles for the intracellular delivery, inducing delay in growth, increased necrosis, fibrosis and reduced cell proliferation in the xenografted tumor in mice [156]. The PNA-mediated targeting of miR-21 resulted in the efficient knockdown of miR-21, inhibition of tumor growth and reduced migration in MCF7 and MDA-MB-231 breast cancer cell lines as well as reduction of tumor growth in nude mice in vivo, giving evidence of the potential therapeutic application of PNA-anti-miR-21 for breast cancer treatment [157].

Very recently, a combined application of PNA-anti-miRNA and miRNA mimics have been developed. A PNA complex formed by folic acid (FA) for the recognition of the folic receptor (FR) on cancer cell membrane, the cationic R9 peptide that allows the self-assembly with the miRNA mimic and the PNA anti-miR-21 (FA-R9-PNA) was assembled with the miRNA mimic miR-34a to form a nanocomplex (PMN-34a/21). The PMN-34a/21 nanocomplex was efficiently delivered in FR-positive HeLa cells and resulted in the upregulation of miR-34a and the concomitant downregulation of miR-21, as well as in the modulation of the two miRNAs downstream target genes, eventually promoting cell apoptosis. This new strategy allows the combined functions of simultaneous upregulation of tumor-suppressive miRNAs by the miRNA mimic, and suppression of oncogenic miRNAs by the PNA-anti-miRNA, resulting in a more effective anticancer treatment [158].

Altogether, these studies revealed that miRNA inhibition by PNA is of great interest for cancer treatment. Considering the versatility of PNA design and chemical modification, it is possible to develop PNA-based strategies to target multiple miRNAs simultaneously or, as recently developed, combine miRNA inhibition and miRNA replacement therapies in a single formulation to enhance the effects. Additionally, their implementation in clinics would open new avenues to non-viral gene therapies.

## 5. Conclusions and Future Perspectives

The deregulation of the immune function in tumor onset and development is so fundamental that in recent years, the bulk of the research in cancer biology has focused on the interaction of the tumor with the immune system, and several newly approved therapeutics are, indeed, immunotherapeutics. This has led to indisputable clinical advances in the care of tumor patients in terms of overall and progression-free survival, and even of remission. For certain tumors, immunotherapies have allowed meeting the goal of chronicity of the diseases and, consequently, of a better control. However, there is still the urgent need for novel and more effective therapeutics in order to contrast the more aggressive forms and the relapses.

Research on breast cancer has allowed significant progress in the prevention and treatment of this neoplasm, however, there are always a number of cases that escape the treatments, which are more aggressive and which progress first to silent micrometastases and then to macrometastases overt to the bone. Unfortunately, for bone metastases there are no curative therapies; in fact, the neoplastic cells that reach the bone are normally resistant to current therapeutic approaches and the only options for these patients are palliative in an attempt to reduce pain and prevent bone destruction. Then, targeting the immune system, by eliminating the blockages put by the tumor cells, could be a favorable approach that appears to be effective even alone and in combination with standard chemotherapy or more advanced treatments to prevent cancer progression and, in the case of breast cancer, bone metastasis.

miRNAs are potentially effective targets within this strategy, since they are able to affect the cell response by controlling at the same time several pathways whose redundancy is the basis of the resistance to chemotherapeutics. Since difficulties in targeting miRNAs, especially in vivo, among the possible approaches here presented, the use of PNA represents a feasible strategy to be tested in the near future.

## Figures and Tables

**Table 1:** Oncogenic miRNAs and tumor-suppressive miRNAs and their role in breast cancer progression.

miRNA	Cells/Tissue	Role	Target	Function	Reference
**Oncogenic miRNAs**					
miR-155	BC and other tumors	im-miRNA	SPI1, Ship1	Increases production of pro-tumor cytokines, reduces expression of activation markers, shifts from M1-like TAM to M2-like TAM	[51]
miR-23a/27a/24-2 cluster	BC	im-miRNA	JAK1, STAT-6, IRF4, PPAR-γ	Modulate macrophage polarization	[52]
miR-195, miR-497	TNBC cells	im-miRNA	PD-L1	Promote tumor progression, inhibit the immune response and promote tumor immune escape	[53]
miR-20a, miR-93, miR-520d, miR-106b, miR-373	prostate, kidney, BC, glioblastoma cells	NK-mediated immune response evasion	MICA/B	Inhibit the NKG2DL-NKG2D pathway, escape from NK-mediated killing	[56,57]
miR-10b	Several cancer cell lines (prostate, colorectal, cervical cancer cells, BC cells)	NK-mediated immune response evasion	MICB	Inhibits NK-mediated cytotoxicity in vitro and contrasts tumor clearance in vivo	[58]
	BC, pancreatic cancer, glioblastoma, other highly metastatic cancers	metastasis formation	TBX5, neurofibromin, KLF4	Promotes proliferation, migration and invasion	[59,60]
			HOXD10	Leads to the upregulation of the pro-metastatic gene RhoC with consequent promotion of tumor cells invasion and metastasis in BC	[61,62]
miR-302c, miR-520c	leukemia and BC cell lines	NK-mediated immune response evasion	MICA/B, ULBP2	Mediate NK-dependent cell killing upon Vitamin D3 treatment	[63]
miR-20a, miR-20b, miR-93, miR-106b	BC cells	NK-mediated immune response evasion	MICA/B, ULBP2	Affect the capacity of NK to recognize and kill tumor cells	[64]
miR-20a	BC stem cells	NK-mediated immune response evasion, metastasis formation	MICA/B	Reduces susceptibility to NK-mediated cell lysis, enhances the metastatic potential	[65]
miR-519a-3p	BC cells	NK-mediated immune response evasion	MICA, ULBP2, TRAIL-R2, caspase-7, caspase-8	Impairs NK-mediated cytotoxicity, confers resistance to apoptosis, regulates tumor progression and evasion from immunosurveillance	[66]
miR-9	BC	metastasis formation	E-cadherin, LIFR	Promotes EMT and metastasis formation through activation of the Hippo signaling pathway	[67,68,69]
miR-200c/141 cluster	TNBC	metastasis formation	SerpinB2 upregulation	Promotes tumorigenesis and metastasis in lungs and lymph nodes in vivo, through overexpression of SerpinB2	[70]
miR-374a	BC	metastasis formation	PTEN, WNT5A	Promotes EMT, induces cell proliferation and metastasis by activating the Wnt/β-catenin pathway	[71]
miR-103/107, miR-630	BC	metastasis formation	Dicer	Promote EMT, migration and metastasis	[72,73]
**Tumor-suppressive miRNAs**					
miR-19a-3p	BC	im-miRNA	Fra-1	Promotes shift from M2- to M1-phenotype of TAMs both in vitro and in vivo, inhibition of metastasis	[54]
miR-240-5p	BC	im-miRNA	PIK3CB	Alters expression of cytokines, remodeling and reprogramming of the tumor microenvironment, promote tumor growth and metastasis in vivo	[55]
miR-3609	BC cells	im-miRNA	PD-L1	Blocks PD-L1 immune checkpoint and sensitize BC cells to adriamycin	[74]
miR-126, miR-335	BC	metastasis suppressor	SOX4, tenascin C (miR-335)	Their restoration in BC cell lines reduces tumor growth and proliferation, inhibits cell invasion, migration and metastasis, inhibit lung and bone metastasis formation in vivo	[75]
miR-126, miR-126*	BC	metastasis suppressor		Prevent recruitment of MSC and monocytes at tumor microenvironment, contrasting tumor cell migration, invasion and metastasis	[76]
miR-200 family, miR-205	BC	metastasis formation	ZEB1, ZEB2	Inhibit EMT, promote metastasis formation in lung and liver in vivo when overexpressed	[77,78]
miR-190	BC	metastasis suppressor	SMAD2	Inhibits EMT and metastasis formation by regulating TGFβ/SMAD2 signaling pathway	[79]
miR-148a	BC	metastasis suppressor	WNT1	Inhibits cell migration, invasion and metastasis by inhibiting the Wnt/β-catenin pathway	[80]
miR-340	BC cells	metastasis suppressor	c-MYC, CTNNB1, ROCK1	Inhibits cell migration, invasion and metastasis by inhibiting the Wnt/β-catenin pathway	[81]
miR-34a, miR-34c	BC	metastasis suppressor	Tgif2	Suppress osteoclastogenesis, bone resorption and bone metastasis	[82]
miR-124	BC cells	metastasis suppressor	IL-11	Suppresses bone metastasis by inhibiting osteoclast progenitor cells differentiation and survival	[83]
miR-30 family	ER−/PR− BC cells	metastasis suppressor	IL-8, IL-11, DKK1, RUNX2, CDH11, CTGF, ITGA5, ITGB3	Inhibits tumor cell invasiveness, restore bone homeostasis in vitro, reduce bone metastasis in vivo	[84]

**Abbreviations.** BC: breast cancer; im-miRNA: immune-modulatory miRNA; TAM: tumor associated macrophages; TNBC: triple negative breast cancer; NK: natural killer cells; EMT: epithelial-mesenchymal transition; MSC: mesenchymal-stem/stromal cells.

## Abbreviations

miRNA	Micro RNA
ER	Oestrogen Receptor
PR	Progesteron Receptor
HER2	Human Epidermal Growth Factor Receptor 2
TNBC	Triple Negative Breast Cancer
MDSC	Myeloid-Derived Suppressor Cell
NK	Natural Killer
SRE	Skeletal-related Event
DTC	Disseminated tumor cell
TIL	Tumor infiltrating lymphocyte
DC	Dendritic Cell
APC	Antigen Presenting Cell
MHC	Major Histocompatibility Complex
TIIC	Tumor-Infiltrating Immune Cell
OS	Overall Survival
TAM	Tumor-Associate Macrophage
PD-L1	Programmed cell Death-Ligand 1
im-miRNAs	immune-modulatory miRNAs
TNF	Tumor Necrosis Factor
MICA	Major Histocompatibility Complex Class I–Related molecule A
MICB	Major Histocompatibility Complex Class I–Related molecule B
ULB1-6	Unique Long 16 Binding Protein 1-6
TRAIL	TNF-related apoptosis-inducing ligand
HDACi	Histone Deacetylase Inhibitors
EMT	Epithelial-Mesenchymal Transition
HOXD10	Homeobox D10
LIFR	Leukemia Inhibitory Factor Receptor
AMO	Anti-miRNA Oligonucleotides
LNA	Locked Nucleic Acid
SMIR	Small Molecule Inhibitors of miRNAs
PNA	Peptide Nucleic Acids
CPP	Cell-penetrating Peptide
PLGA	poly(lactic-co-glycolic acid)
